# Chemerin regulates normal angiogenesis and hypoxia-driven neovascularization

**DOI:** 10.1007/s10456-021-09818-1

**Published:** 2021-09-15

**Authors:** Cyrine Ben Dhaou, Kamel Mandi, Mickaël Frye, Angela Acheampong, Ayoub Radi, Benjamin De Becker, Mathieu Antoine, Nicolas Baeyens, Valérie Wittamer, Marc Parmentier

**Affiliations:** 1 WELBIO and I.R.I.B.H.M, Université Libre de Bruxelles, Campus Erasme, 808 route de Lennik, B-1070 Brussels, Belgium; 2 Physiologie de la Reproduction et des Comportements, University of Tours, INRA Val-de-Loire UMR-85, CNRS UMR-1247, Tours, France; 3 Cardiology Department, Erasme Hospital, Université Libre de Bruxelles, Route de Lennik 808, B-1070 Brussels, Belgium; 4 Laboratoire de Physiologie et Pharmacologie, Université Libre de Bruxelles, Campus Erasme, 808 route de Lennik, B-1070 Brussels, Belgium

**Keywords:** CMKLR1, ChemR23, Retinal angiogenesis, Tumoral neoangiogenesis, Hind-limb ischemia model, Oxygen-induced retinopathy

## Abstract

Chemerin is a multifunctional protein initially characterized in our laboratory as a chemoattractant factor for leukocyte populations. Its main functional receptor is CMKLR1. We identified previously chemerin as an anti-tumoral factor inhibiting the vascularization of tumor grafts. We show here that overexpression of bioactive chemerin in mice results in a reduction of the density of the retinal vascular network during its development and in adults. Chemerin did not affect vascular sprouting during the post-natal development of the network, but rather promoted endothelial cell apoptosis and vessel pruning. This phenotype was reversed to normal in CMKLR1-deficient mice, demonstrating the role of this receptor. Chemerin inhibited also neoangiogenesis in a model of pathological proliferative retinopathy, and in response to hind-limb ischemia. Mechanistically, PTEN and FOXO1 antagonists could almost completely restore the density of the retinal vasculature, suggesting the involvement of the PI3-kinase/AKT pathway in the chemerin-induced vessel regression process.

**Supplementary Information:**

The online version contains supplementary material available at 10.1007/s10456-021-09818-1.

## Introduction

Angiogenesis is an essential and complex process, by which new blood vessels grow from preexisting ones, as a tightly regulated response of endothelial cells (EC) to a combination of pro- and anti-angiogenic factors [1–3]. The establishment of a functional blood vessel networks is initiated by the formation of a primitive vascular plexus, from which new blood vessels sprout, coordinately expand, and branch. Redundant vessel branches are then selectively removed through vessel regression, a process called pruning [4, 5]. This process is necessary for establishing the hierarchic vessel patterning during angiogenesis, and the removal of redundant or damaged vascular branches [6]. Mechanistically, vessel regression is regulated by hemodynamics and numerous signaling pathways [6].

Chemerin is a secreted protein of 16 kDa [7] initially characterized in our laboratory as a chemoattractant factor for immature myeloid and plasmacytoid dendritic cells (DC), macrophages, and natural killer (NK) cells [8]. It was later described as an adipokine regulating lipogenesis and lipid metabolism [9]. Chemerin is produced by many cell types, including fibroblasts, adipocytes, hepatocytes, and various epithelial cells as an inactive precursor, prochemerin. Prochemerin is processed into bioactive chemerin by the proteolytic removal of the last 6 or 7 C-terminal residues of the precursor [10]. While the main functional receptor of chemerin is CMKLR1 (also called ChemR23 or chemerin_1_), GPR1 (chemerin_2_), and CCRL2 were also described as chemerin receptors, with respectively limited and no signaling properties [11, 12]. CMKLR1 is coupled to the G_i/o_ family of G proteins and to β-arrestin recruitment, and leads to inhibition of adenylate cyclase, release of intracellular Ca^2+^ and phosphorylation of ERK1/2 [11, 13, 14]. CMKLR1 is expressed by leukocyte populations (macrophages, DCs, NK cells) but also by endothelial cells, adipocytes, and smooth muscle cells [15–17].

We and others have identified chemerin as an anti-tumoral factor in different mouse models [18–20]. We recently proposed that, in mouse cancer graft models, the anti-tumoral activity of chemerin does not involve the recruitment of leukocyte populations but is mediated by the inhibition of the vascularization of the tumors, an effect mediated through CMKLR1 [21].

Hypoxia is the major trigger of tumor neovascularization. Hypoxic conditions promote the stabilization of the hypoxia-inducible transcription factor (HIF), which results in the upregulation of many genes, including those encoding vascular endothelial growth factors (VEGF) and their receptors. These factors stimulate the release of proteolytic enzymes by endothelial cells, as well as their proliferation and migration, leading to vascular sprouting [22]. Besides the essential role of angiogenesis during development, pathologic conditions in adults may promote neovascularization as a result of an acute or chronic hypoxic environment in tissues [23]. This hypoxia-triggered neovascularization response may be insufficient, such as in ischemic diseases, or rather excessive in proliferative retinopathies, such as the retinopathy of prematurity (ROP), in which aberrant neoangiogenesis is a common cause of vision loss and blindness [23–26].

Following the identification of chemerin as a negative regulator of angiogenesis in tumors, we investigated the consequence of chemerin overexpression and invalidation of its receptors in other physiological or pathological contexts involving neoangiogenesis or vascular remodeling. The development of the vascular retinal network in mice is a very robust model allowing to follow the different steps of the angiogenesis process. We observed in this system that chemerin favors the pruning of blood vessels once formed, as well as the apoptosis of endothelial cells. This results in a reduction of the density of the retinal vascular network, which persists until adulthood. Similar anti-angiogenic properties were also observed in a model of oxygen-induced retinopathy, mimicking the retinopathy of prematurity in human, and in the hind-limb ischemia model that investigates the vascular remodeling response to acute ischemia.

## Results

### Chemerin regulates blood vessel density in the postnatal retina in an EC sprouting-independent manner

In order to investigate the role of chemerin on physiological angiogenesis *in vivo*, we used a transgenic mouse model described earlier, in which a bioactive form of chemerin is overexpressed by skin keratinocytes, under the control of the keratin 5 gene promoter (K5-Chem model) [19]. In these mice, the levels of chemerin immunoreactivity and bioactivity are strongly elevated in the blood, affecting therefore all tissues in the body. The post-natal development of the vascular network in the retina of these mice was explored. This model is suitable to analyze the different phases of angiogenesis, including vessel sprouting, maturation, and remodeling/pruning [27].

**Fig. 1:**
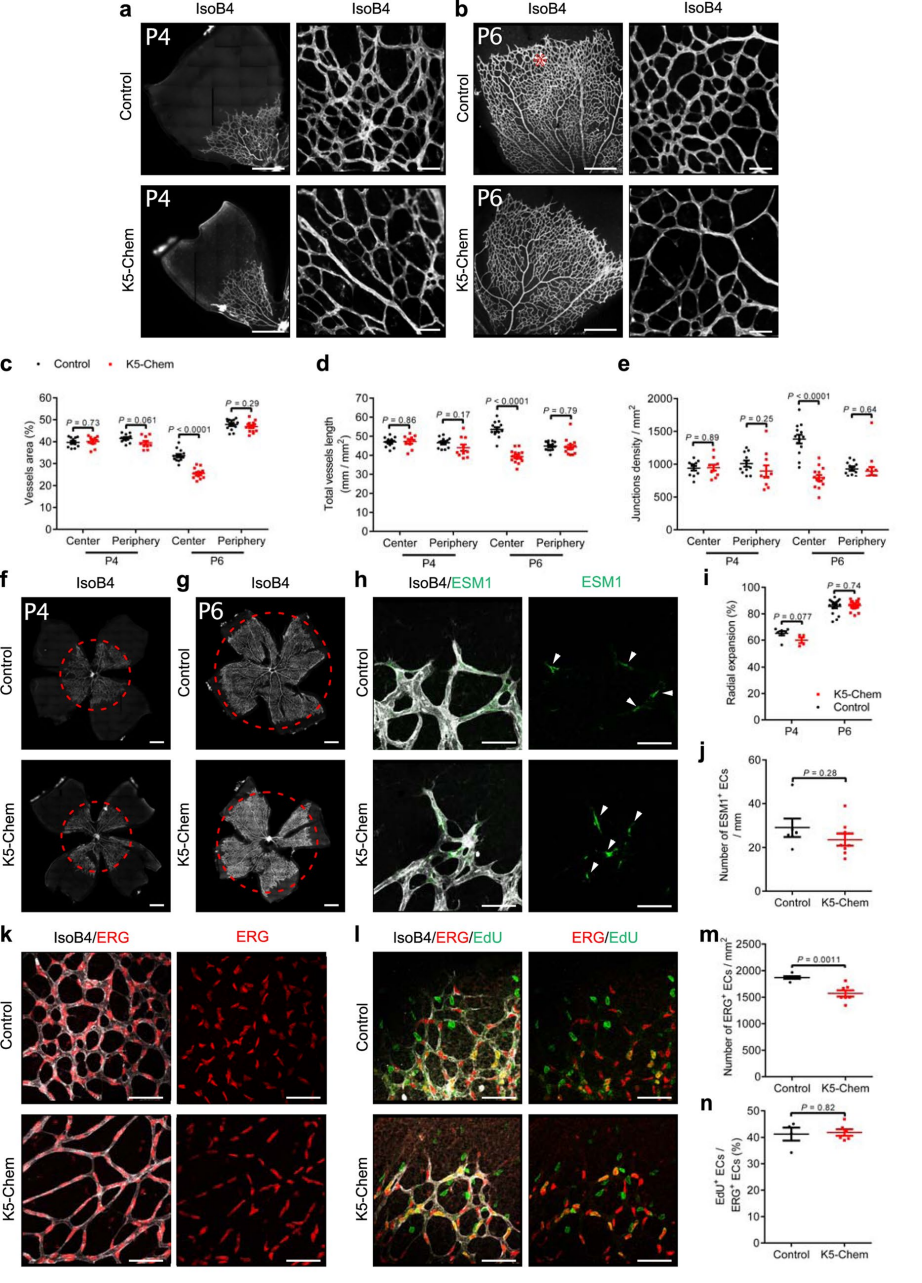
Overexpression of chemerin decreases the density of the developing vessel retinal network, without affecting sprouting. **a, b** Representative images of retinas from mice overexpressing chemerin (K5-Chem) and their wild-type controls at post-natal days 4 (P4) and 6 (P6), stained with isolectin B4 (IsoB4). Scale bars: 500 μm in left panels, 50 μm in right panels. **c**–**e** Quantification of the vessels area, total vessels length, and junctions density, normalized for the surface, in the peripheral and central parts of the plexus (*n* = 11 for P4 controls, *n* = 10 for P4 K5-Chem, *n* = 13 for P6 controls, *n* = 14 for P6 K5-Chem). **f, g** Representative images of whole mount retinas from K5-Chem and control mice at P4 and P6, stained with IsoB4. Scale bars: 500 μm. **h** Representative confocal images of the angiogenic front of the retinal network at P6, stained with IsoB4 and for ESM1. White arrows point to endothelial tip cells expressing ESM1. Scale bars: 50 μm. **i** Radial expansion of the vascular network relative to the retina radius in K5-Chem and control mice (*n* = 7 for P4 controls, *n* = 4 for P4 K5-Chem, *n* = 16 for P6 controls, *n* = 18 for P6 K5-Chem). **j** Quantification of the number of ESM1^+^ tip cells per mm of network front in K5-Chem (*n* = 8) and control mice (*n* = 6). **k** Representative confocal images of the remodeling zone of the retinal network from WT and K5-Chem mice at P6, stained with IsoB4 and for ERG. Scale bars: 50 μm. **l** Representative confocal images of the periphery of the retinal network from control and K5-Chem mice at P6, stained with isoB4 and for EdU and ERG. Scale bars: 50 μm. **m** Density of ERG^+^ ECs in the remodeling plexus of K5-Chem (*n* = 7) and control mice (*n* = 6). **n** Quantification of EdU^+^ ECs among ERG^+^ cells at the network periphery in K5-Chem (*n* = 6) and control mice (*n* = 4). *P* values versus controls by two-tailed unpaired Student’s *t*-test. All data are shown as mean ± SEM. Each point represents 1 animal

The retinas of mice overexpressing chemerin (K5-Chem) showed a reduced density of the vascular network at postnatal day 6 (P6), while no differences were seen at postnatal day 4, when compared to control littermates (Fig. 1a, b). At P6, chemerin overexpression significantly decreased the relative vessels area (Fig. 1c), the total vessels length per surface unit (Fig. 1d) and the density of branch points (Fig. 1e) in the central part of the capillary plexus, compared to control mice. However, at the periphery of the network, in the area located immediately behind the leading tip cells, (Fig. 1b, asterisk), the same parameters were not affected (Fig. 1c, d, e).

The extent of the network, as determined by the distance between the central artery and the periphery of the superficial vascular plexus was not modified either at P4 or P6 (Fig. 1f, g, red line, and Fig. 1i). In line with this observation, we did not detect changes in the number of cells labeled for endothelial cell-specific molecule 1 (ESM1), a tip cell marker [28, 29] (Fig. 1h, j). The decrease in vessel density was correlated with a decrease in the density of EC, as determined by the nuclear labeling for the EC-specific transcription factor ERG (Fig. 1k, m). We also evaluated the proliferation rate of endothelial cells at P6 by labeling nuclei for the ETS-related gene (ERG) product and measuring the percentage of cells labeled by 5-ethynyl-2’-deoxyuridine (EdU), injected into mice 2.5 h before sacrifice. The proportion of EdU^+^ cells among ERG^+^ cells was about 40 %, with no difference between mice overexpressing chemerin and their controls (Fig. 1l, n). These data suggested that the phenotype did not result from changes in vessel sprouting or endothelial cell proliferation, pointing instead toward increased regression during the remodeling phase.

**Fig. 2:**
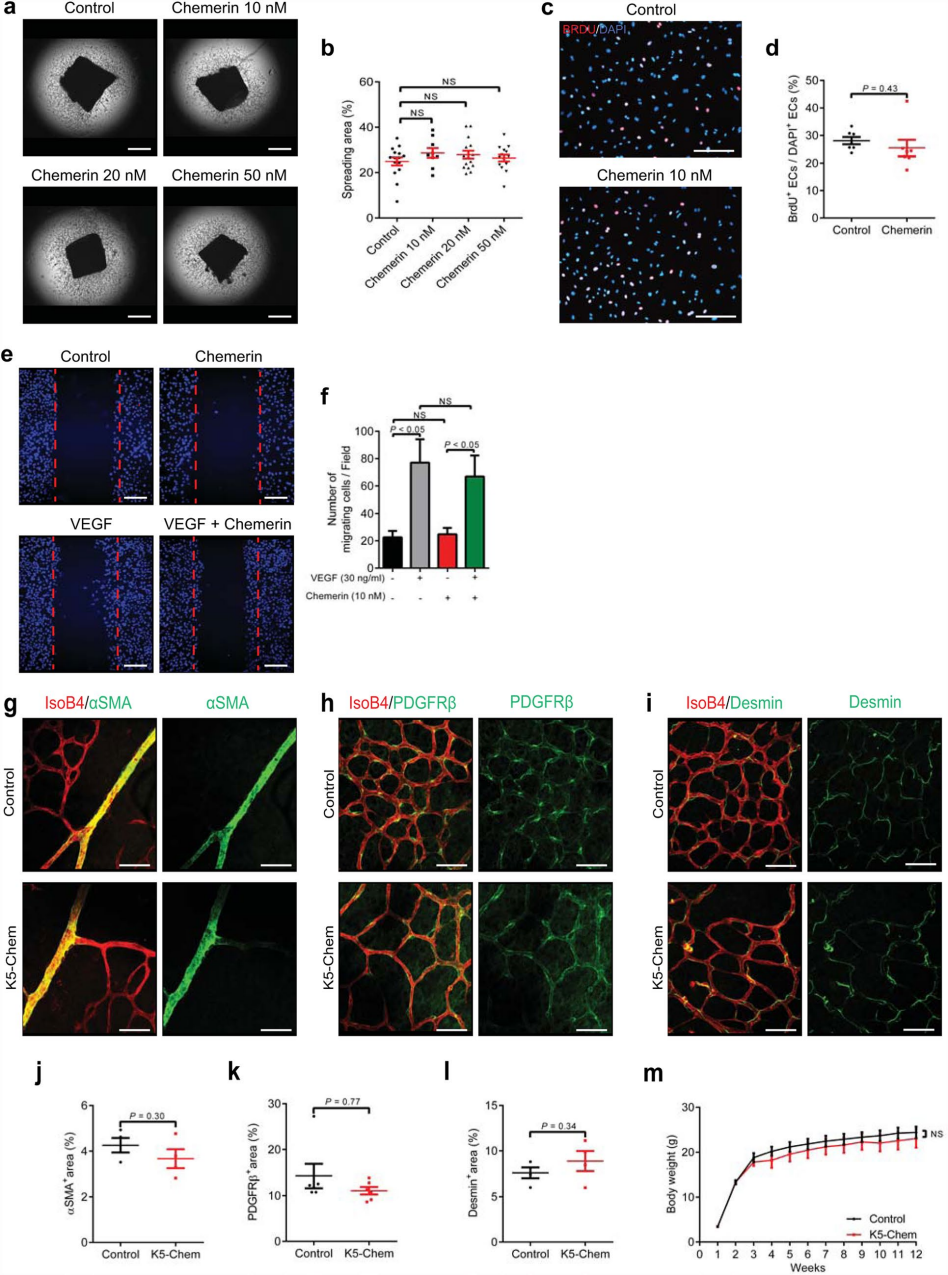
Chemerin does not affect vessel sprouting nor pericyte coverage. **a** Representative images of the capillary network sprouting from aortic ring explants collected from WT mice and treated or not with 10, 20, or 50 nM chemerin. Scale bars: 500 μm. **b** Quantification of the area of capillary outgrowth (*n* = 15 for controls, *n* = 9, 15, and 14 for, respectively, 10, 20, and 50 nM chemerin). *P* values versus control by one-way ANOVA with Tukey’s post hoc test. *NS* not significant. **c, d** Representative images and quantification of BrDU^+^ cells in a culture of HUVECs treated or not with 10 nM chemerin and stained with DAPI (*n* = 7). **e, f** Representative images and quantification of the migration of HUVEcs treated or not with VEGF and/or 10 nM chemerin and stained with DAPI in a scratch assay (*n* = 3). Scale bars: 200 μm. *P* values by one-way ANOVA with Tukey’s post hoc test. *NS* not significant. **g**–**i** Representative confocal images of the pericyte coverage on vessels from P6 retinas. The retinas were co-stained with IsoB4 and for either α-SMA, PDGFRβ, or desmin as pericyte markers. Scale bars: 50 μm. **j**–**l** Quantification of the area stained for α-SMA (*n* = 4), PDGFRβ (*n* = 6), and desmin (*n* = 4) relative to the vascular area (%). **m** Weight curves of control and K5-Chem mice between 1 and 12 weeks after birth (*n* = 12). *P* values versus control by 2-tailed unpaired Student’s t-test. All data are shown as mean ± SEM. Each point represents 1 animal or one well

To characterize further the influence of chemerin on the EC phenotype and try to delineate which steps of the angiogenesis process are affected in chemerin-overexpressing mice, we investigated EC sprouting *ex vivo* using the mouse aortic ring assay. Aortic rings were incubated with 30 ng/ml VEGF, in the presence or not of various concentrations of mouse recombinant chemerin (10, 20, and 50 nM). In this model, chemerin did not increase nor decrease the extent of vessel sprouts at any of the concentrations tested (Fig. 2a, b) thereby supporting that chemerin does not affect the sprouting step of angiogenesis. We also tested the effect of chemerin on the proliferation and migration of human umbilical vein endothelial cells (HUVEC). The cells were cultured for 24 h in the presence or absence of 10 nM chemerin and exposed for the last 3 h to BrdU. Chemerin did not affect, either positively or negatively, the ratio of BrdU^+^ cells (Fig. 2c, d), demonstrating that chemerin has no effects on the proliferation rate of endothelial cells. In a scratch wound-healing assay, chemerin did not modify the migration of HUVECs promoted by VEGF-A (Fig. 2e, f).

We investigated further whether a defect in pericyte coverage might result from chemerin activity. Co-staining of endothelial cells by isolectin B4 (IsoB4), and of pericytes by antibodies directed at three markers, α-SMA (Fig. 2 g, j), PDGFR (Fig.  2h, k), and desmin (Fig. 2i, l), did not reveal obvious changes in the coverage of vessels by pericytes in retinas of K5-Chem mice, suggesting that this crucial step in vessel maturation is not affected [30].

Altogether, these data suggest a role of chemerin during developmental angiogenesis, possibly at the level of vessel remodeling rather than vessel sprouting and maturation. As previously reported [19], however, mice overexpressing chemerin develop normally to adulthood and appear grossly normal despite the alteration of developmental angiogenesis (Fig. 2m).

### Chemerin promotes microvessel regression and EC apoptosis

**Fig. 3:**
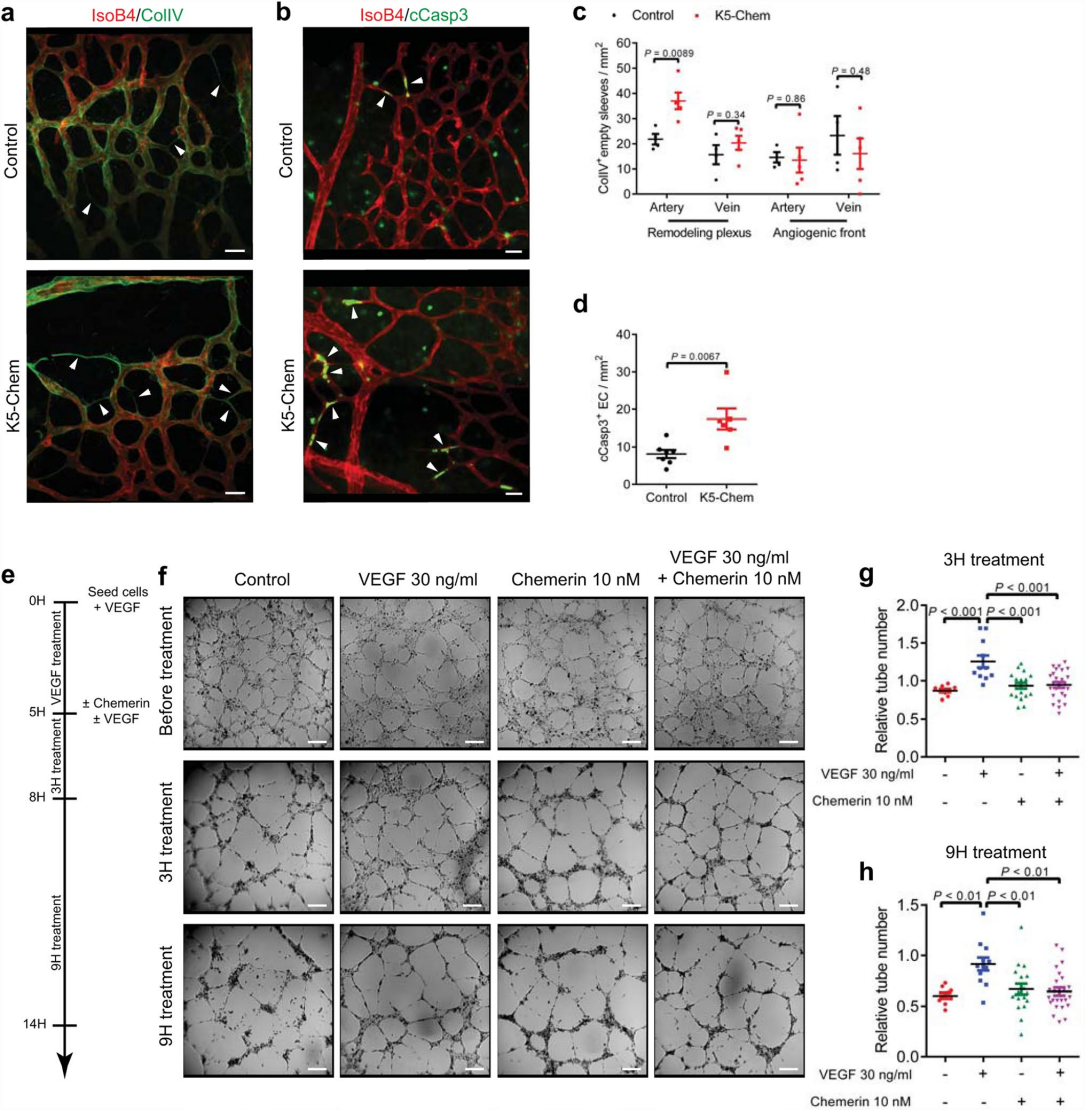
Overexpression of chemerin promotes vessel regression and endothelial cell apoptosis.** a** Representative confocal images of the remodeling plexus of P6 retinas collected from control or K5-Chem mice, stained with IsoB4 and for collagen IV (ColIV). White arrows point to ColIV^+^ IsoB4^−^ empty sleeves. Scale bars: 20 μm. **b** Representative confocal images of the remodeling plexus of P6 retinas collected from control or K5-Chem mice, stained with IsoB4 and for cleaved caspase-3 (cCasp3). Apoptotic cells are indicated by white arrows. Scale bars: 20 μm. **c** Quantification of empty sleeves in the remodeling plexus and the angiogenic zone, in the vicinity of arteries or veins (*n* = 5). **d** Quantification of cCasp3^+^ IsoB4^+^ cells in the remodeling plexus of K5-Chem (*n* = 6) and control mice (*n* = 7). **e** Diagram depicting the experimental timeline of the tube assay using HUVECs, allowing formation of tubes for 5 h in the presence of VEGF, and measuring the evolution of the network after 3 or 9 h in the presence or absence of 30 ng/ml VEGF and/or 10 nM chemerin. **f** Representative images of HUVEC tubes at 3 and 9 h in control conditions (*n* = 9) or after treatment with 10 nM chemerin (*n* = 19), 50 ng/ml VEGF (*n* = 11), or the combination of both (*n* = 25). Scale bars: 200 μm. **g, h** Quantification of the number of tubes 3 and 9 h after chemerin and/or VEGF treatment, normalized to the tube number before treatment (pool of 6 independent experiments). For **c**, **d**, *P* values versus controls by 2-tailed unpaired Student’s t-test. For **g**, **h**, *P* values versus controls by one-way ANOVA with Tukey’s post hoc test. All data are shown as mean ± SEM. Each point represents one animal or one well

During the regression phase of the retinal network development, redundant blood vessels constrict, and ECs retract. The basal lamina of the regressed vessels can be visualized as empty sleeves following staining for type IV collagen (ColIV) [6]. The number of empty sleeves was significantly higher in the retina of K5-Chem mice at post-natal day 6, in the more mature region of the plexus proximal to the optic nerve, and particularly in the vicinity of arteries (Fig. 3a, c, arrowheads), suggesting a role of chemerin in the control of the switch between vessel maintenance and vessel regression. No significant difference was seen in the vicinity of veins in the capillary plexus, neither close to the angiogenic front of the growing retinal network (Fig. 3c). As both EC apoptosis and migration to other vessel branches may contribute to the remodeling process [6, 31], we investigated whether overexpression of chemerin affected the rate of endothelial cell death. Interestingly, we observed a significant increase in the number of cleaved caspase-3^+^ cells (cCasp3^+^) (Fig. 3b, d, arrowheads), suggesting that chemerin action favors apoptosis of ECs, contributing thereby to the physiological remodeling process and supporting vessel pruning.

We then evaluated the effect of chemerin on HUVEcs in the tube formation assay. Chemerin did not influence the formation of the tubes either positively or negatively (data not shown). However, when chemerin (10 nM) was added on pre-established tube networks formed in the presence of VEGF (Fig. 3e), it promoted a faster regression of the network after 3 and 9 h, counteracting the effects of VEGF (Fig. 3f–h). These data support a destabilization role of chemerin on established endothelial cell tubes.

### CMKLR1 mediates the anti-angiogenic effects of chemerin

**Fig. 4:**
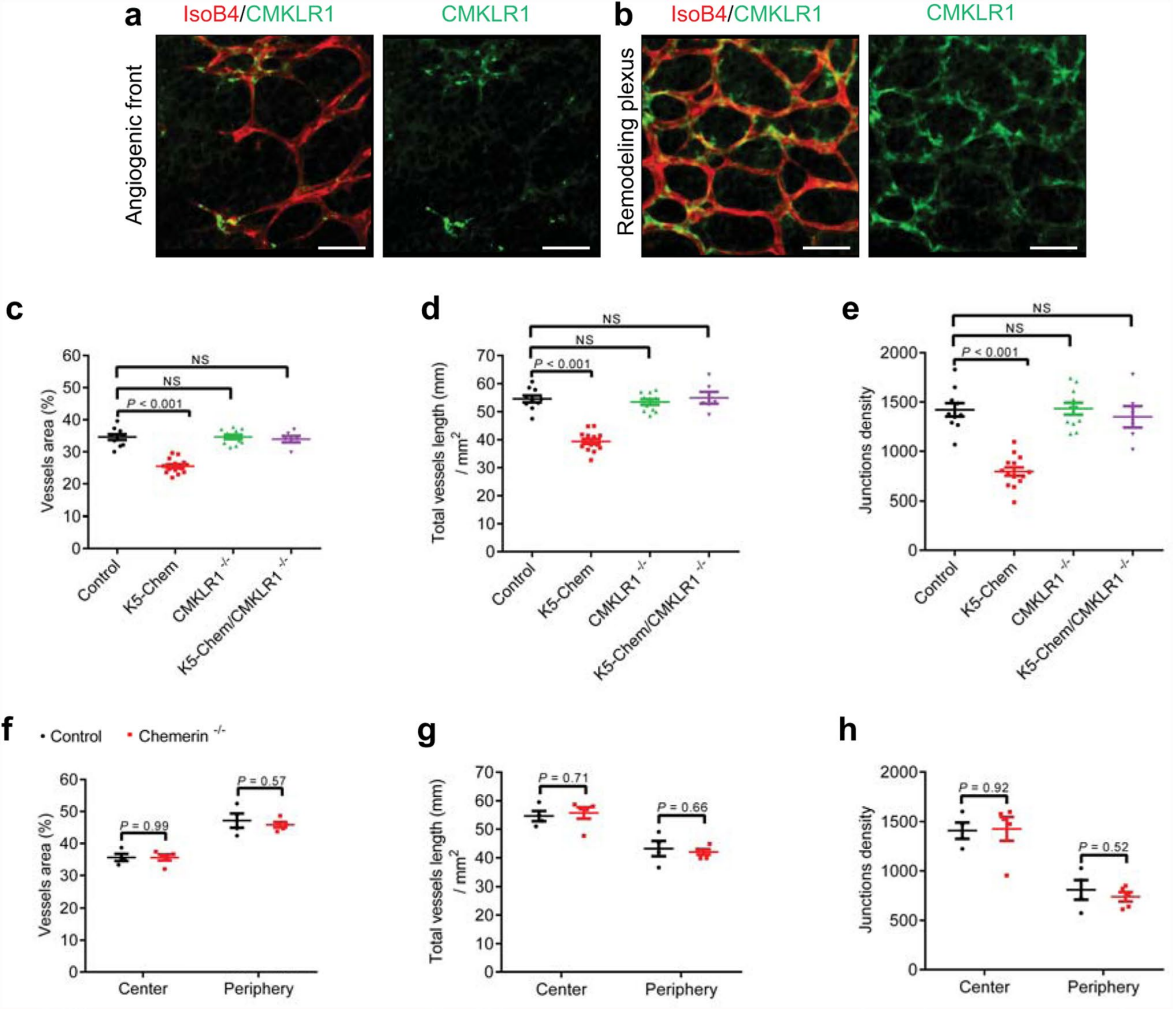
CMKLR1 mediates the anti-angiogenic effects of chemerin.** a, b** Representative images of the retinas from P6 control mice, stained with IsoB4 (red) and for CMKLR1 (green) in the sprouting zone and remodeling plexus. Scale bars: 50 μm. **c**–**e** Quantification of the vessels area, total vessels length, and junction density in the remodeling plexus of P6 retinas of control mice and mice overexpressing chemerin and/or invalidated for CMKLR1 (n = 10 for controls, n = 14 for K5-Chem, n = 11 for CMKLR1^−/−^, *n* = 6 for K5-Chem/CMKLR1^−/−^). *P* values versus control by one-way ANOVA with Tukey’s post hoc test. **f**–**h** Quantification of the vessels area, total vessels length, and junctions density in the remodeling and proliferative zones of the P6 retina from control (*n* = 4) and chemerin^−/−^ mice (*n* = 5). P values versus control by 2-tailed unpaired Student’s *t*-test. All data are shown as mean ± SEM. Each point represents 1 animal

CMKLR1 is the main functional receptor of chemerin. Consistent with published data on tip-cell-enriched genes [32], CMKLR1 immunostaining decorated endothelial cells from the remodeling plexus and the angiogenic front in postnatal day 6 retinas (Fig. 4a, b). In order to determine the role of CMKLR1 in the phenotype of mice overexpressing chemerin, we used a mouse line genetically invalidated for this receptor that was described previously [15]. The vessels area, the total vessels length, and the junctions density at P6 were not affected in K5-Chem/CMKLR1^−/−^ pups compared with control littermates (Fig. 4c–e), indicating that the effects of chemerin on angiogenesis are entirely mediated by CMKLR1. Also, no changes were seen in CMKLR1^−/−^ pups, nor in chemerin^−/−^ pups (Fig. 4f–h) as compared to WT control mice, suggesting that the endogenous production of chemerin does not play a major role during the development of the retinal network in normal conditions.

**Fig. 5:**
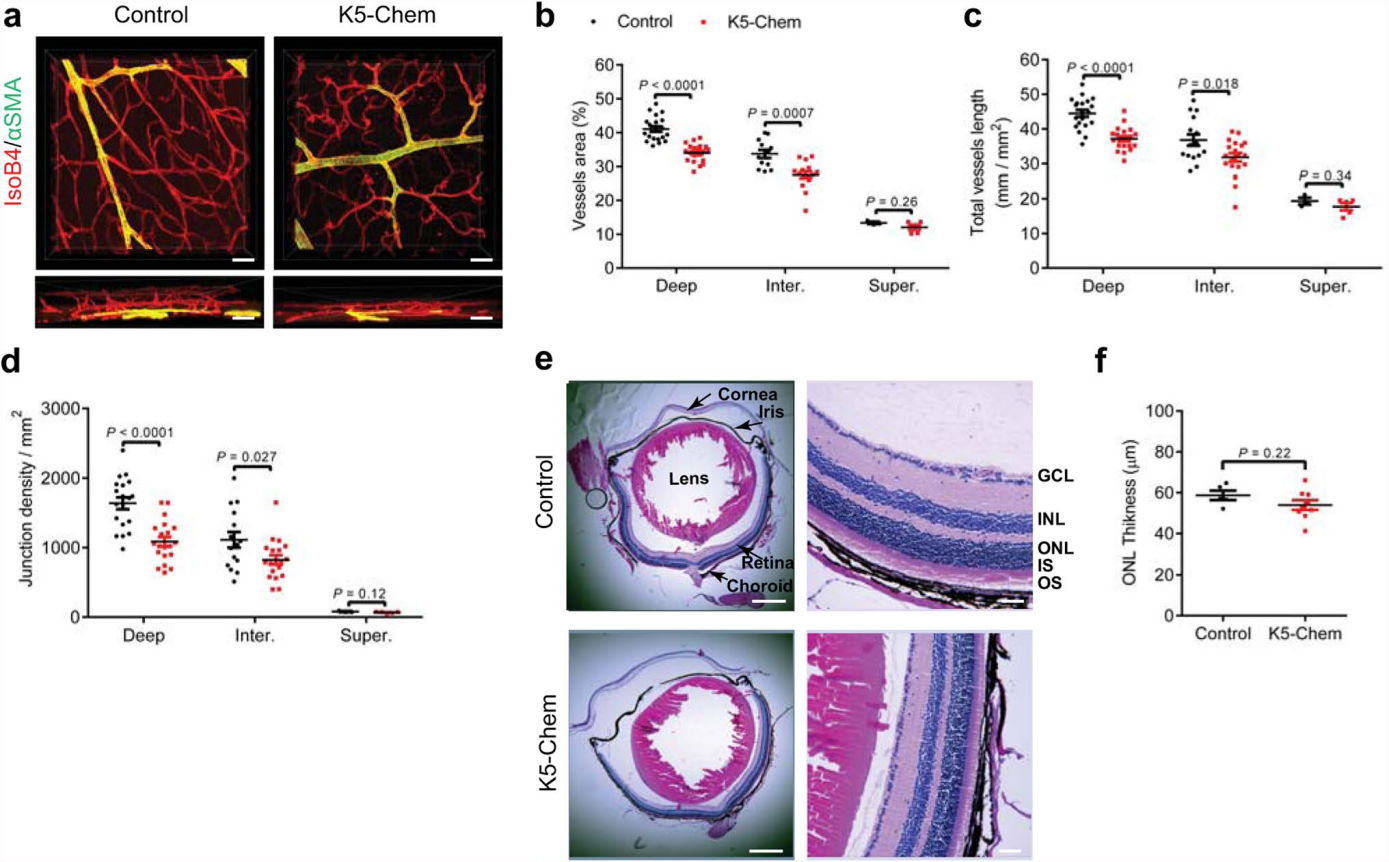
The lower vascular density in the retina is maintained in adult mice overexpressing chemerin. **a** Representative 3D images of the vessel architecture in the retina of control and K5-Chem adult mice. Scale bars: 20 μm. **b**–**d** Quantification of the vessels area, total vessels length, and junctions density in the deep (*n* = 27 for controls, *n* = 37 for K5-Chem), intermediate (*n* = 15 for controls, *n* = 19 for K5-Chem), and superficial layer (*n* = 3 for controls, *n* = 5 for K5-Chem) of adult mice. Each point represents 1 field of view. **e** Representative images of H&E-stained sagittal ocular sections from 8-week-old control and K5-Chem mice. Scale bars: 500 μm in left panels, 50 μm in right panels. **f** ONL thickness was quantified in K5-Chem (*n* = 8) and control mice (*n* = 5). *GCL* ganglion cell layer, *INL* inner nuclear layer, *IS* inner segment, *ONL* outer nuclear layer, *OS* outer segment. *P* values versus controls by 2-tailed unpaired Student’s *t*-test. All data are shown as mean ± SEM

The mature vascular network of adult mouse retinas was analyzed at 6 to 8 weeks. A lower density of vessels was observed in adult K5-Chem mice in the intermediate and deep layers, as compared to controls, while no differences were seen in the superficial layer (Fig. 5a–d; Supplementary Movies 1, 2). The vessels of the deep plexus and those connecting the intermediate and deep layers also appeared more tortuous in K5-Chem mice, as compared to the relatively straight vessels seen in control mice. These observations indicate the absence of compensatory mechanisms counteracting the consequences of chemerin overexpression during the late stages of retina development and growth. The ocular structure and retinal cell layers appeared otherwise normal in hematoxylin/eosin-stained sections of adult K5-Chem mice (Fig. 5e, f).

Altogether, these data show that overexpression of bioactive chemerin during retinal development results in a reduction of the vascular density originating early during retinal angiogenesis as a result of increased vessel pruning and EC apoptosis. These effects are mediated by CMKLR1, and their consequences persist throughout development and adulthood.

### Chemerin decreases pathological angiogenesis in a model of oxygen-induced retinopathy

**Fig. 6:**
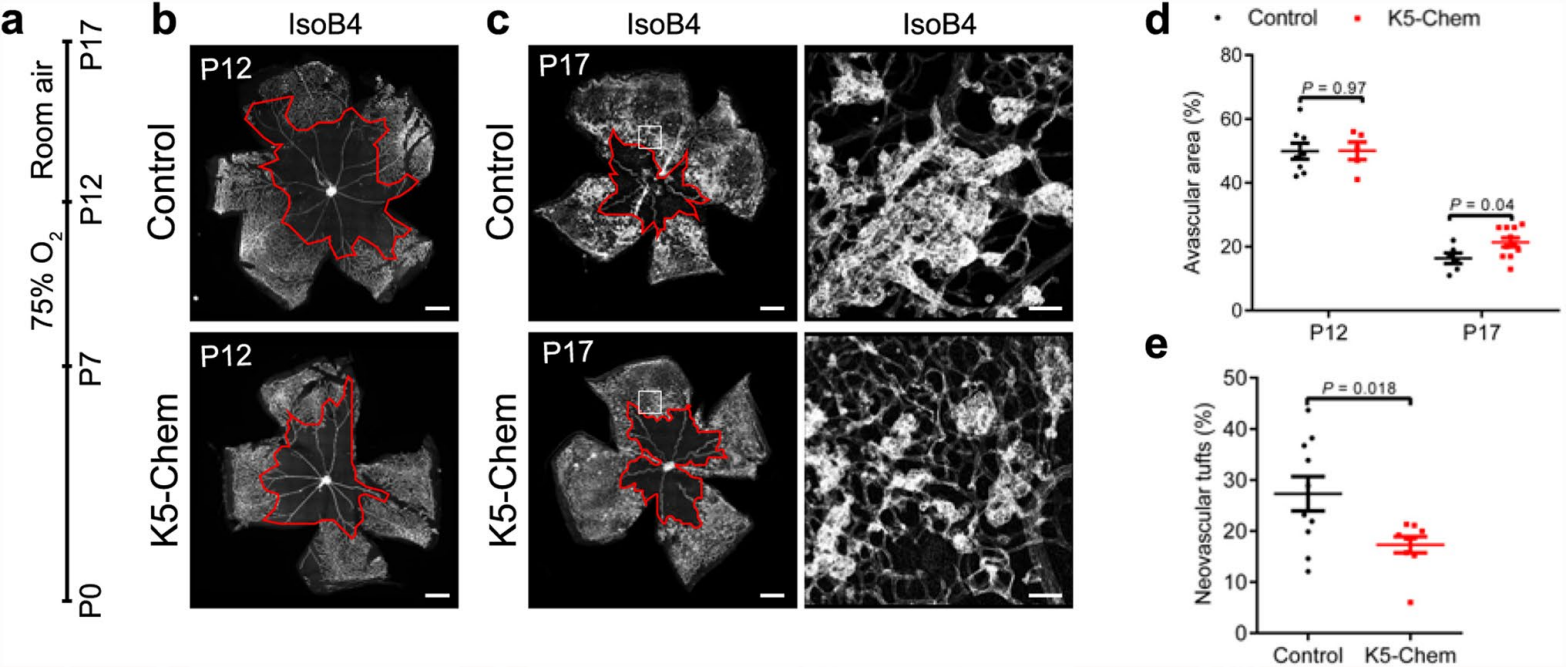
Chemerin reduces neovascularization in a model of oxygen-induced retinopathy (OIR). **a** Diagram depicting the experimental timeline of the OIR protocol. Pups were placed in 75 % oxygen (hyperoxia) from P7 to P12, then placed back under normal oxygen conditions (normoxia) until P17. **b**, **c** Representative images of the retinal vasculature stained with IsoB4 in control and K5-Chem mice at P12 and P17. Scale bars: 500 μm in left panels, 50 μm in right panels. The red line marks the limits of the avascular area. **d** Quantification of the avascular area in control and K5-Chem mice at P12 and P17 (*n* = 8 for P12 controls, *n* = 5 for P12 K5-Chem, *n* = 6 for P17 controls, *n* = 11 for P17 K5-Chem). **e** Quantification of the tufts area in control (*n* = 10) and K5-Chem (*n* = 9) mice. P values versus controls by 2-tailed unpaired Student’s t-test. All data are shown as mean ± SEM. Each point represents 1 animal

To determine whether CMKLR1 agonists might be relevant in ocular neovascular diseases, we used a mouse model of oxygen-induced retinopathy (OIR) that closely recapitulates the two phases of retinopathy of prematurity (ROP) [33]. Mouse pups were exposed to high oxygen levels (75%) from P7 to P12, to induce vessel obliteration (Fig. 6a). The return to normal oxygen levels after p12 results in a severe retinal hypoxia, increasing endothelial cell proliferation and promoting exuberant pathological neovascularization, as well as a gradual replacement of the lost vessels in central retina [34, 35]. The hyperoxia-induced vessel obliteration in the central capillary network was similar in K5-Chem and control mice at P12, suggesting that chemerin did not significantly affect this phase of the model (Fig. 6b, d). However, analysis of the retinal vasculature at P17 showed that pathological neovascularization, quantified by measuring the area of neovascular tufts [36], was reduced in the retinas of K5-Chem mice, as compared to controls (Fig. 6c, e). The avascular area at P17 was also slightly larger in K5-Chem pups compared to WT littermates (Fig. 6c, d). These data support the concept that chemerin prevents disease progression in a model of OIR, resembling ROP, reducing the severity of tuft formation without compromising the newly formed vasculature.

### Chemerin inhibits ischemic tissue recovery and angiogenesis in a model of hind-limb ischemia

**Fig. 7:**
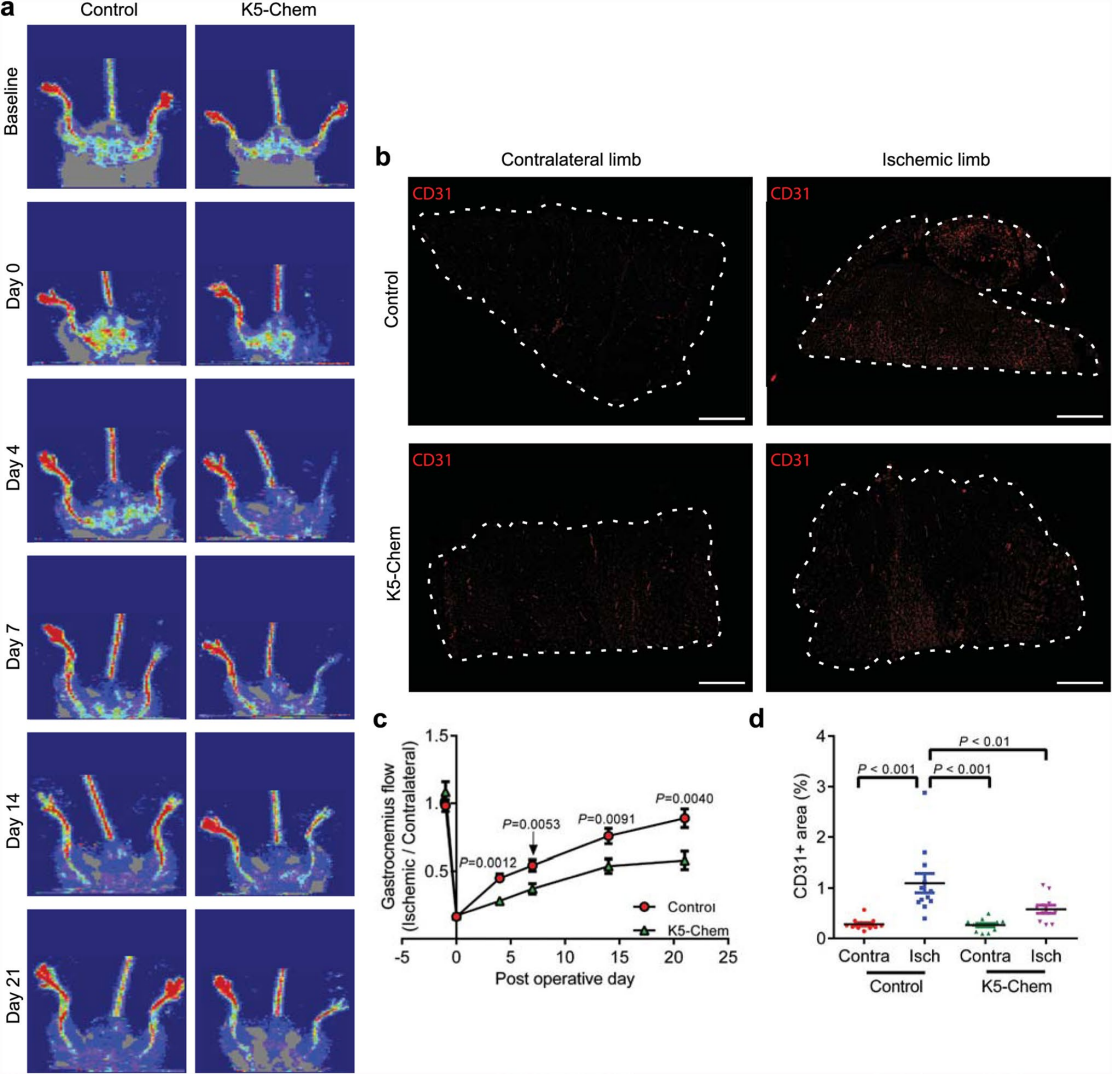
Chemerin inhibits ischemic tissue recovery and angiogenesis in a model of hind-limb ischemia.** a** Representative laser Doppler images showing delayed reperfusion in the right hind limb of K5-Chem mice at days 4, 7, 14, and 21 following femoral artery ligation, as compared to control mice. **b** Representative images of gastrocnemius muscle sections stained for CD31. Scale bars: 1 mm. **c** Quantification of the distal flow in the gastrocnemius muscle by Doppler measurement in K5-Chem and control mice (*n* = 15). *P* values versus control by 2-tailed unpaired Student’s *t*-test. **d** Quantification of CD31^+^ area in the ischemic and contralateral limb of control and K5-Chem mice at day 21 (*n* = 12 for controls, *n* = 13 for K5-Chem, pool of 3 independent experiments). *P* values by one-way ANOVA with Tukey’s post hoc test. All data are shown as mean ± SEM. Each point represents 1 animal

The functional role of chemerin in hypoxia-driven angiogenesis was further studied in the hind-limb ischemia model (HLI), in which neovascularization is induced by the acute disruption of blood supply in the hind limb and the resulting tissue ischemia and hypoxia [37]. After ligature of the right femoral artery, the recovery of blood flow in the paw was investigated up to 21 days later by laser Doppler imaging, using the left side as control. While WT mice exhibited nearly complete restoration of the blood flow (∼80%) after 21 days, K5-Chem mice could only attain ∼50% of the pre-HLI perfusion ratio (Fig. 7a, c). We also investigated the vessel density in the gastrocnemius muscle, by labeling endothelial cells with an anti-CD31 antibody. We observed that the capillary density was significantly lower in the injured limb of K5-Chem mice (Fig. 7b, d). These data reveal that chemerin inhibits the neovascularization process and the overall functional recovery in a model of acute ischemia.

### Chemerin destabilizes blood vessels in tumor models

**Fig. 8:**
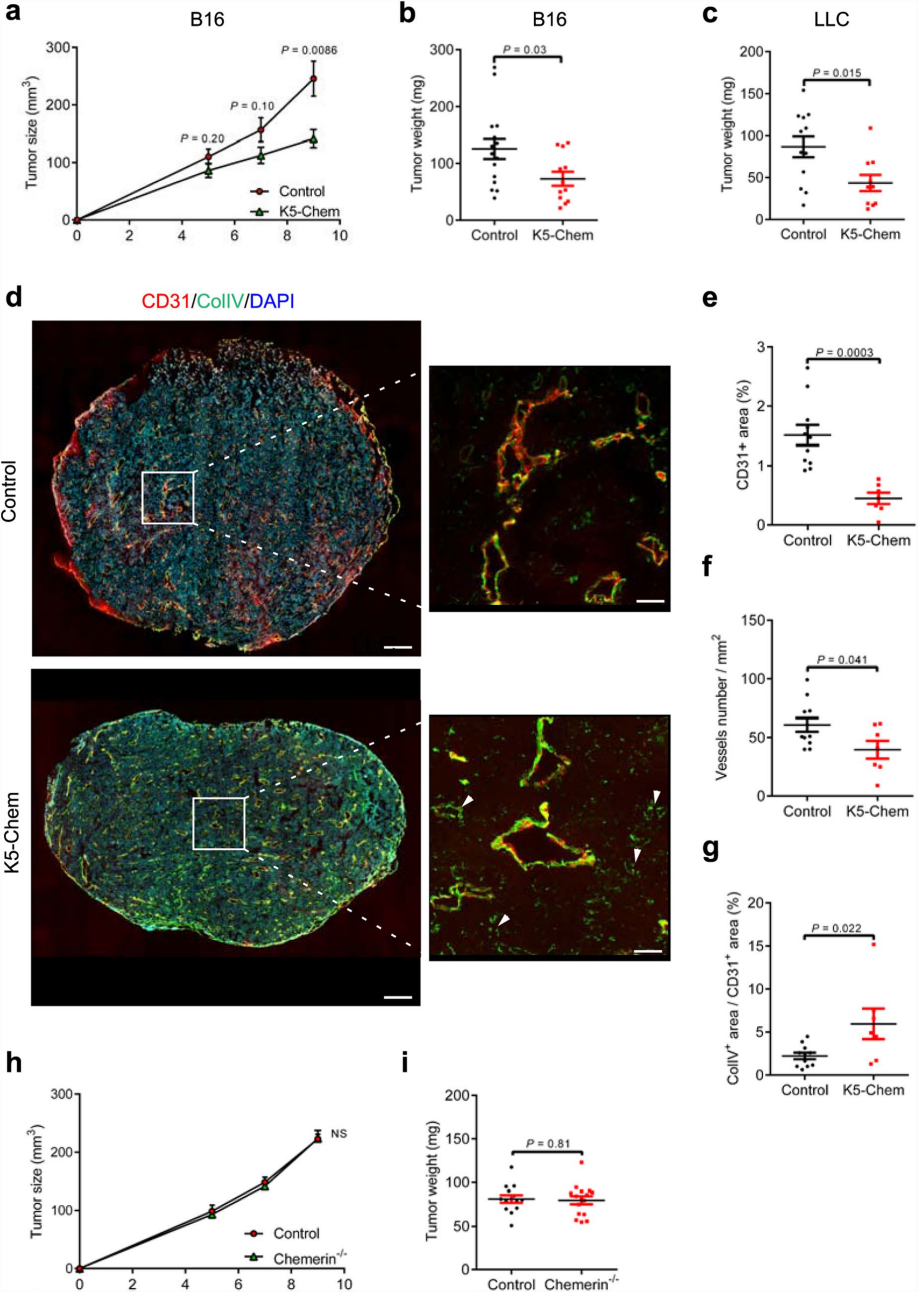
Chemerin favors tumor vessel regression. **a** 1.10^6^ B16-F0 cells were implanted into the left flank of control (*n* = 15) and K5-Chem (*n* = 12) mice (in 3 independent experiments). The tumor size was monitored with a caliper until day 9. **b** The weight of the tumors was measured following sacrifice at day 9. **c** 1.10^6^ LLC cells were implanted into the left flank of mice and the weight measured after sacrifice at day 13 (*n* = 12 for controls, *n* = 10 for K5-Chem). **d** Immunofluorescence staining for CD31 and ColIV were performed on cryosections of B16 tumors collected from control and K5-Chem mice at day 9. Scale bars: 500 μm in left panels, 100 μm in right panels. **e**–**g** Quantification of the CD31^+^ area, vessels number, and ColIV^+^ area relative to the CD31^+^ vascular area (%) in B16 tumors (*n* = 11 for controls, *n* = 7 for K5-Chem). **h** Control and chemerin^−/−^ mice were grafted with B16 cells and the tumor size was measured. **i** The weight of the tumors was measured following sacrifice at day 9 (*n* = 14 for controls, *n* = 16 for chemerin^−/−^). *P* values versus control by 2-tailed unpaired Student’s *t*-test. All data are shown as mean ± SEM. Each point represents 1 animal

Chemerin was shown to display anti-tumoral properties in several mouse models [18, 20]. In a recent study, we reported that these properties could be attributed to an inhibitory effect on the vascularization of tumors, resulting in necrotic cell death [21]. We investigated further whether this anti-angiogenic effect in a tumoral context could be mediated by a destabilization of neovessels. We used B16-F0 melanoma and Lewis lung carcinoma (LLC), two models reported to progress in an angiogenesis-dependent manner [38, 39]. As reported previously [21], grafting B16-F0 or LLC cells in K5-Chem mice resulted in smaller tumors than in control mice (Fig. 8a–c). Staining for the endothelial specific marker CD31 also confirmed the lower density of endothelial cells in B16 tumors harvested at day 9 from mice over-expressing chemerin (Fig. 8d–f). Staining for type IV collagen identified a higher area of ColIV-stained basal lamina associated with blood vessels in tumors from K5-Chem mice as compared to controls (Fig. 8d). In control tumors, ColIV staining lined closely the endothelial cells and had a smooth appearance, while in tumors from K5-Chem mice, ColIV was more widely distributed around the vessels, and the ratio of ColIV^+^ to CD31^+^ areas was increased (Fig. 8g). The same observations were made on 3D reconstitutions of cleared LLC tumors (Supplementary Movies 3, 4). Such enlargement of ColIV staining around blood vessels in LLC tumors was previously attributed to a deregulation of blood vessel stability [40]. These data confirm in a tumoral model that chemerin favors regression of vessels resulting from neoangiogenesis. Chemerin invalidation had however no influence on B16 tumor growth (Fig. 8h, i).

### The activity of chemerin involves the PTEN-AKT-FOXO1 axis

**Fig. 9:**
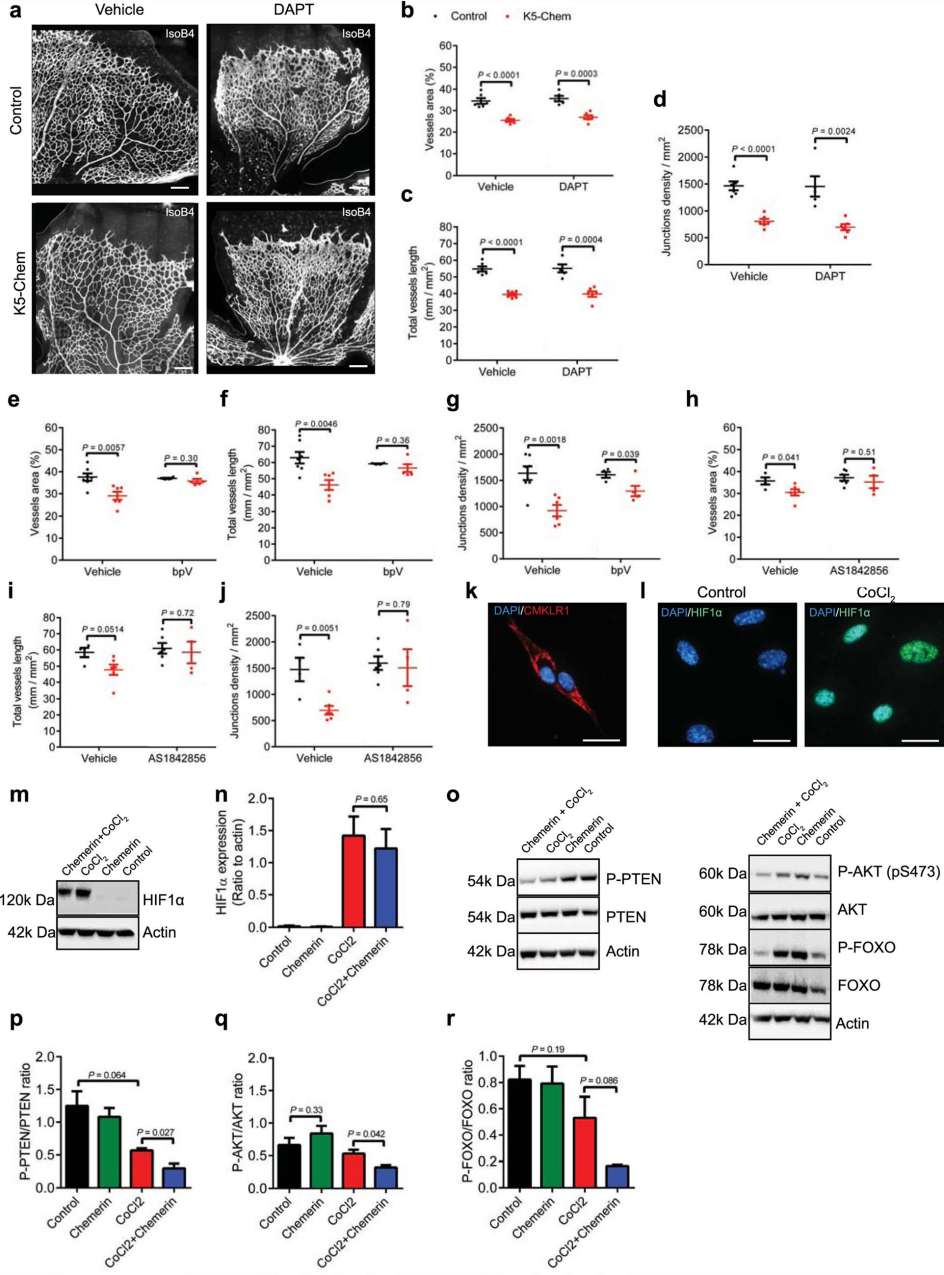
Chemerin activity involves the PTEN-AKT-FOXO1 axis.** a** Representative images of retinas of P6 control and K5-Chem mice, injected intraperitoneally with DAPT or vehicle, after staining with IsoB4. Scale bars: 200 μm. **b**–**d** Quantification of the vessels area, total vessels length, and junctions density in the remodeling plexus of these retinas (*n* = 6 for controls vehicle, K5-Chem vehicle and K5-Chem DAPT, *n* = 5 for controls DAPT). **e**–**g** Quantification of the vessels area, total vessels length, and junctions density in the remodeling plexus of retinas from control and K5-Chem mice injected with bpV(HOpic) or vehicle (*n* = 7 for controls vehicle, *n* = 6 for K5-Chem vehicle, *n* = 4 for controls bpV, *n* = 5 for K5-Chem bpV). **h**–**j** Quantification of the vessels area, total vessels length, and junctions density in the remodeling plexus of retinas from control and K5-Chem mice injected with AS1842856 (AS) or vehicle (*n* = 4 for controls vehicle and K5-Chem AS, *n* = 6 for K5-Chem vehicle and controls AS). **k** Representative image of HUVECs stained for CMKLR1. Scale bar: 20 μm. **l** Representative images of HUVECs treated or not with CoCl_2_ and stained by DAPI and for HIF1a. Scale bars: 20 μm. **m** Representative Western blot for HIF1a detection in HUVECs treated or not with CoCl_2_ and/or 10 nM chemerin. **n** Quantification of HIF1a normalized to actin. **o** Representative Western blots of HUVECs treated or not with CoCl_2_ and/or 10 nM chemerin. **p**–**r** quantification of the phosphorylation ratios of PTEN, AKT, and FOXO1. *P* values versus controls by 2-tailed unpaired Student’s *t*-test. All data are shown as mean ± SEM. Each point represents 1 animal

We next investigated the signaling pathways involved in EC regression that could be altered in K5-Chem ECs. Vessel regression or inefficient angiogenesis has been linked to hyper-activation of Notch signaling [41–43]. To test whether this pathway is involved in chemerin-induced vessel regression, we treated mice with the γ-secretase inhibitor DAPT. The resulting inhibition of Notch signaling in control mice led to increased EC density and sprouting at the angiogenic front (Fig. 9a). However, we still observed a decrease in vessels area, total vessels length, and junctions density in the remodeling plexus of K5-Chem mice (Fig. 9b–d), suggesting that the effect of chemerin is not mediated through the Delta/Notch pathway.

Signaling downstream of CMKLR1 was reported to result in phosphatase and tensin homolog (PTEN) activation in different cell types [44, 45], and the PI3K-AKT cascade is known to promote endothelial cell survival. We therefore hypothesized that enhanced PTEN activity in endothelial cells, resulting from chemerin stimulation, might contribute to the regression of neovessels. To test this hypothesis, pups were treated twice with the specific PTEN inhibitor bpV(HOpic) [46] before analysis of their retina at P6. The inhibitor did not modify the parameters of the vascular network in control mice, but completely reversed the effect of chemerin in K5-Chem mice (Fig. 9e–g). Forkhead box protein O1 (FOXO1) is well established as a negative regulator of EC metabolism, proliferation and survival [47, 48], and is inactivated by phosphorylation by AKT [49, 50]. As for PTEN inhibition, the treatment of pups by the FOXO1-specific inhibitor AS1842856 (AS), did not modify the structure of the vascular network in control P6 mice. In K5-Chem pups, however, the vessels area, total vessels length, and junctions density were reversed to control levels following the treatment (Fig. 9 h–j).

These results *in vivo* suggest that stimulation of CMKLR1 in endothelial cells results in the activation of PTEN, a downregulation of the PI3K-AKT pathway, and enhanced FOXO1 activity, favoring EC apoptosis and vessel regression. This hypothesis was tested further on primary cultures of human umbilical vascular endothelial cells (HUVECs). CMKLR1 expression by these cells was confirmed by immunofluorescence (Fig. 9k). To mimic the effects of hypoxia, the cells were treated with 100 µM CoCl_2_, promoting the stabilization of HIF1α, and a sharp accumulation of the protein after 24 h (Fig. 9l, m). Chemerin had no effect on this HIF1α increase (Fig. 9n).

The amount and phosphorylation state of PTEN, AKT, and FOXO proteins, as representative elements of the PI3K/AKT pathway and its activation, were investigated by Western blotting. In “normoxic” conditions, treatment by 10 nM chemerin for 24 h did not affect the total level of each protein (quantification relative to a-actin not shown), nor their phosphorylation ratio. Treatment by 100 mM CoCl_2_ (“hypoxic” conditions) for 24 h did not modify total protein levels but decreased significantly the proportion of Ser380/Thr382/Thr383-phosphorylated and inactive form of PTEN. There was also a tendency toward a decrease of the Ser473-phosphorylated (active) form of AKT and the phosphorylated and inactive form of FOXO1, without reaching statistical significance (Fig. 9o–r). Treatment with chemerin in these hypoxic conditions amplified the decrease in the phosphorylation state for all three proteins, reflecting a further activation of PTEN and FOXO1, and the inactivation of AKT. These results on HUVECs confirm therefore the ability of chemerin to inactivate the PI3K-AKT pathway through an activation of PTEN. These effects were however detectable only in conditions mimicking hypoxia, a context in which neovascularization occurs in vivo.

## Discussion

Chemerin is a multifunctional protein. As a chemoattractant factor for various leukocyte populations, including macrophages, dendritic cells, and natural killer cells, it is endowed with pro- or anti-inflammatory properties according to the context and the disease model [10]. CMKLR1 is the main functional receptor of chemerin. Besides leukocytes, it is expressed by endothelial cells and smooth muscle cells, suggesting a range of potential roles in angiogenesis and the control of blood flow [12]. Several studies have proposed a positive action of chemerin on the angiogenesis process mainly *ex vivo* on HUVEC-based models, but also *in vivo* [16, 51, 52]. A contribution of chemerin as a vasoconstrictor molecule contributing to the control of vascular tone was proposed [53].

Chemerin was also described to display anti-tumoral properties in a number of cancer models, as well as pro-tumoral effects in some others [54]. In previous studies, we described the anti-tumoral properties of chemerin in several models of cancer [19, 21]. These effects were attributed to an inhibition of tumoral neoangiogenesis, leading to hypoxia and necrosis. These recent observations are in direct opposition with the conclusions of studies reported above, claiming that chemerin has pro-angiogenic effects both *in vitro* and *in vivo* [16, 51, 52].

For the study of these tumoral models, we generated a transgenic mouse line in which a bioactive form of chemerin is expressed in the basal cells of the skin epithelium, resulting in a significant rise in circulating levels of the protein [19]. During the initial analysis of this mouse line, we did not identify obvious changes in the development of the animals, in the histology of tissues and organs, nor in the distribution of leukocyte populations in the skin or in primary and secondary lymphoid organs. In the present work, we used this genetic model of bioactive chemerin overexpression to explore the potential effect of chemerin on the development of the vascular network of the retina, and the resulting structure of this network in adults. Retinal angiogenesis became indeed a classical model to study neoangiogenesis *in vivo*, and to delineate the pathways involved in its regulation.

The retinal vascular network develops from the central vein and artery. A first network of capillaries develops by radial expansion toward the periphery of the retina during post-natal days 3 to 7. At the periphery of this growing network, classical neoangiogenesis occurs, driven by relative hypoxia and VEGF production, sprouting tip cells leading stalk cells in a collective migration paradigm. The central remodeling zone of the network is characterized by an adaptation to the local demand of oxygen, usually resulting in the regression of unnecessary vessels and a simplification of the plexus. In later days (P7–P15), sprouting vessels invade the outer parts of the retina, forming the deep and intermediate layers seen in adults. During the vessel remodeling phase, local differences in blood flow and shear stress determine which vessel segments are pruned away [55, 56]. This pruning process is driven essentially by EC migration [55–57], but EC apoptosis contributes to the removal of non-perfused segments during vessel maturation and the establishment of optimal vessel diameter in capillaries [58].

In the present study, we demonstrate that chemerin can contribute to the regulation of angiogenesis. We confirmed that CMKLR1, the main receptor of chemerin, is expressed by retinal endothelial cells, including during the development of the network. We show that chemerin does not affect the progression of the vascular network toward the retina periphery, the sprouting and proliferation of ECs, nor the coverage of vessels by pericytes. Instead, chemerin promotes the regression of vascular branches in the remodeling zone of the plexus and EC apoptosis, as demonstrated by the presence of empty sleeves and activated caspase 3-positive cells. In contrast to other situations affecting the early vascular network of the retina [59, 60], these changes were not compensated later in life, and adult mice were still characterized by a less dense vascular network, at least in the intermediate and deep layers. The overall organization of the retina did not appear to be affected by these modifications in the vascular network. All these changes were totally reversed in mice invalidated for CMKLR1, demonstrating the role of this receptor in the process, and eliminating the possibility of indirect or artefactual activities of chemerin through other and unidentified mechanisms.

The destabilization effects of chemerin could be reproduced *in vitro*, on HUVECs in the tube formation assay. While chemerin did not affect tube formation, it counteracted the effects of VEGF on the stability of the tubes, promoting their regression. In our hands, chemerin had no effects on neovessel sprouting in the aorta ring assay, nor on migration or proliferation of HUVECs in culture. It should be noted however that we reported recently a strong inhibitory effect of chemerin on the extent of the capillary network formed by HUVECs in the bead sprouting assay [21].

These observations contrast sharply with previous reports claiming a positive effect of chemerin on the angiogenesis process. One of these studies reported a positive but moderate effect of chemerin on the formation of tubes in a coculture of ECs and fibroblasts [51]. Curiously, these effects were only observed for chemerin concentrations well below the EC_50_ for CMKLR1, while the concentration near the EC_50_ (10 ng/ml, around 0.5 nM) was ineffective. Another report described positive effects of chemerin on the proliferation of HUVECs and the formation of tubes in Matrigel, although the active concentrations in these two assays were quite discordant [16]. A later report described positive effects of chemerin *in vivo*, in Matrigel plugs and the mouse corneal angiogenesis assay, as well as in the rat aortic ring assay, while confirming the previously reported activities on EC migration, proliferation, and tube formation *in vitro* [52]. We cannot at this point reconcile these observations with our own data, as similar assays were used with very different outcomes.

Following the observation that chemerin can regulate the physiological process of neoangiogenesis during the development of retina, we investigated whether similar observations could be made in models of pathological neoangiogenesis. In the oxygen-induced retinopathy model, we observed indeed a slight delay in the recovery of a “normal” vascular network, but a significant reduction in pathological angiogenesis, reflected by the extent of vascular tufts. The OIR model simulates closely the retinopathy of prematurity, a blinding disease observed in some premature infants placed on oxygen support therapy to compensate their insufficient lung development [61]. OIR also mimics some aspects of proliferative diabetic retinopathy. Consequently, acting through CMKLR1 might be considered as a therapeutic avenue in these pathological situations involving excessive or inappropriate angiogenic responses in the retina. In the hind-limb ischemia model [62], a delay in the recovery of distal blood flow was observed in mice overexpressing chemerin, and this functional defect was correlated with the reduction in the number of vessels observed in the gastrocnemius muscle collected from the lesioned side. In complement to our observations on tumors reported elsewhere [21], we observed in LLC and B16 tumor grafts from chemerin-overexpressing mice a fuzzy and extended labeling for collagen IV around tumor vessels. Similar observations have been related to the lack of stability of the neovessels in other studies [40], suggesting that the reduction in tumor vascularization might be similarly due to decreased stability promoting vessel regression.

It should be noted that invalidation of the CMKLR1 gene or chemerin gene RARRES2 did not modify the development or adult structure of the retinal vascular network. This indicates that chemerin does not contribute significantly to the regulation of angiogenesis in normal conditions. Bioactive chemerin is generated as the result of proteolytic activity in the context of inflammatory reactions, tissue remodeling, and repair processes. Efficient concentrations are therefore likely not reached during the physiological development of the retina and other organs. However, the situation might be very different in neoangiogenesis taking place in the frame of pathological situations, such as acute or chronic inflammatory diseases, ischemia and reperfusion injuries, or tissue reconstruction following traumatic insults. All these situations involve the activation of protease cascades, which result in the activation of prochemerin. In all these pathological situations, chemerin might therefore contribute to the regulation of the neoangiogenesis process, preventing excessive angiogenesis in some cases, but also possibly impairing the recovery of adequate tissue perfusion in others. Our observations regarding the protective role of chemerin in the oxygen-induced retinopathy model, and the delayed recovery of perfusion in the hind-limb ischemia model support indeed this hypothesis.

We finally started to delineate the signaling cascades involved in the activity of chemerin on endothelial cells. CMKLR1 is coupled to the G_i/o_ family of heterotrimeric G proteins, and the recruitment of β-arrestins, leading to the inhibition of cAMP accumulation, intracellular calcium release, and the activation of MAPK cascades including ERK1/2 and p38, as well as the PI3K/AKT pathway. Activation of ERK1/2, p38, and AKT have been reported in endothelial cells as a response to chemerin, and linked to the described effects on proliferation, migration, and survival [16, 52]. None of these pathways is an obvious candidate as a negative regulator of vessel stability. We therefore tested the potential implication of alternative pathways not necessarily linked to CMKLR1 but known to regulate vessel pruning. One of the candidates was the Delta/Notch pathway, but its inhibition by a γ-secretase inhibitor did not abrogate the effects of chemerin on the retinal network. Another candidate was FOXO1, described as a critical rheostat of vascular expansion [47, 48]. Endothelium-specific knockout of FOXO1 causes hyper-branching of retinal vasculature, while enhancing its activity reduces EC metabolism and proliferation and promotes apoptosis, thereby reducing vascular morphogenesis. FOXO1 is well known to be negatively regulated by the PI3K/AKT pathway, its phosphorylation impairing its nuclear translocation and hence its activity on transcription [49, 50]. Interestingly, chemerin was previously reported to increase PTEN activity in hepatocellular carcinoma cells, by disrupting a direct interaction with CMKLR1 leading to ubiquitination and degradation of the protein [44]. Another report described a transcriptional upregulation of PTEN promoted by chemerin in prostate and sarcoma tumor lines, mediated by the serum response factor (SRF) and early response 1 (EGR-1) transcription factors [45]. Although these studies do not deal with endothelial cells and describe quite different routes toward PTEN activation, they prompted us to investigate PTEN as a possible upstream regulator of FOXO1 and angiogenesis. This hypothesis was tested by treating pups with selective inhibitors and investigating the consequences on retinal angiogenesis. Inhibitors of PTEN or FOXO1 did not modify the structure of the retinal network in control animals but restored a normal density in chemerin-overexpressing pups. These data suggest that a significant part of the effects of chemerin on endothelial cells *in vivo*, leading to their apoptosis and to vessel regression, is mediated through an activation of PTEN, the inhibition of the PI3K/AKT pathway, and a release of the FOXO1 activity.

These observations *in vivo* were complemented by *in vitro* experiments on HUVECs, confirming that chemerin can decrease the phosphorylation of PTEN, increasing its activity [63, 64], counteracting the activity of AKT, and preventing FOXO1 inactivation. All these observations were however made only in conditions mimicking hypoxia, and the total level of PTEN in the cells was unchanged, in contrast to what was described in tumor cell lines [44, 45].

Altogether, our data point toward a role of chemerin as a negative regulator of the PI3K-AKT pathway, particularly in hypoxic conditions in which neoangiogenesis occurs. This leads to an activation of the FOXO1 transcription factor, resulting in a modification of the balance between endothelial cell survival and neovessel stabilization on one side, and EC apoptosis and vessel regression on the other. These data suggest therapeutic applications of CMKLR1 agonists mimicking the effects of chemerin, with potential benefit in pathological situations involving inappropriate or excessive neoangiogenesis, including solid tumors, retinopathy of prematurity, diabetic retinopathy, and potentially arthritis in which destruction of the cartilage by the neoangiogenesis pathway triggered by inflammation plays a central role.

## Methods

### Mouse lines

C57BL/6J mice were purchased from Janvier. Mice overexpressing chemerin in keratinocytes under the control of the keratin K5 promoter (K5-Chem model) were described previously [19]. The CMKLR1 knockout mouse line was described previously [15]. The chemerin knockout mouse line (C57BL/6 N-*Rarres*2^*tm1*(*KOMP*)*Vlcg*^/MbpMmucd) was obtained from the Mutant Mouse Resource and Research Center at University of California at Davis. All genetically modified mouse lines were bred on the C57BL/6J background. Mice were maintained in a specific pathogen-free facility with environmental enrichment and unlimited access to food and water. Experimental animals were used between day 2 and 10 weeks of age. Transgenic or knockout mice and their respective controls were littermates in all settings. Altogether, 419 mice were used in this study.

### Antibodies and reagents for fluorescence microscopy and Western blotting

For fluorescence microscopy, the following primary antibodies were used: rabbit monoclonal anti-ERG (1:500, Abcam, ab110639), eFluor 660-conjugated mouse monoclonal anti-α-smooth muscle actin (α-SMA, 1:100, Invitrogen, 50-9760-82), rabbit polyclonal anti-cleaved caspase-3 (1:400, Cell Signaling, 9661), rabbit polyclonal anti-mouse collagen IV (1:400, Bio-Rad, 2150−1470), goat polyclonal anti-desmin (1:100, R&D Systems, AF3844), goat polyclonal anti-mouse PDGFRβ (1:100, R&D Systems, AF1042), mouse monoclonal anti-BrdU (1:100, BD Biosciences, 347580), PE-conjugated rat monoclonal anti-mouse CD31 (1:200, eBioscience, 12-0311-83), goat polyclonal anti-mouse ESM1 (1:100, R&D Systems, AF1999), rabbit monoclonal anti-HIF1α (1:100, Cell Signaling, 36169), mouse monoclonal anti-CMKLR1 (1:100, Santa Cruz Biotechnology, sc374570). Secondary antibodies used in immunofluorescence were Alexa Fluor 488-conjugated donkey anti-rabbit IgG, Alexa Fluor 488-conjugated chicken anti-goat IgG, Cy3-conjugated donkey anti-mouse IgG (1:400, Life Technologies and Jackson Laboratories). Alexa Fluor 647-conjugated isolectin GS-IB_4_ (IsoB4, 1:500, I32450, Invitrogen) was used to label endothelial cells and 4′,6-diamidino-2-phenylindole dihydrochloride (DAPI, 1 µg ml^− 1^, Life Technologies, D1306) for staining nuclei.

Primary antibodies used for Western blotting were rabbit polyclonal anti-AKT (1:3000, Cell Signaling, 9272), rabbit monoclonal anti-phospho-AKT (Ser473, 1:3000, Cell Signaling, 4060), rabbit monoclonal anti-FoxO1 (1:3000, Cell Signaling, 2880), rabbit polyclonal anti-phospho-FoxO1 (Thr24)/FoxO3a (Thr32) (1:3000, Cell Signaling, 9464), rabbit monoclonal anti-PTEN (1:3000, Cell Signaling, 9559), rabbit monoclonal anti-phospho-PTEN (Ser380/Thr382/383, 1:3000, Cell Signaling, 9549), rabbit monoclonal anti-HIF1a (1:2000, Cell Signaling, 36169), and rabbit polyclonal anti-a1 actin (1:3000, Sigma, A2066). The secondary antibody was a horseradish peroxidase-conjugated anti-rabbit IgG (H + L) (1:3000, Cell Signaling Technology, 7074).

### HUVEC cell culture and BrdU incorporation assay

HUVECs from a pool of donors were purchased from Lonza and used between passages 2 and 6. They were grown in EBM-2 medium containing EGM supplements (Lonza, CC-3121 and CC-4133) and 10% FBS. To assess the effects of chemerin on signaling cascades, we starved HUVECs 3 h in EBM-2 supplemented with 0.5% FBS and treated them with 10 nM chemerin and/or 100 µM CoCl_2_ for 24 h. For the BrdU incorporation assay, HUVECs were plated on fibronectin-coated glass coverslips (10 µg/mL, Sigma, F1141), starved for 3 h in EBM containing 0.5% FBS. Recombinant human chemerin (R&D Systems, 2324-CM) was then added at the concentration of 10 nM, and 21 h later, 0.1 mg/ml BrdU (Invitrogen, B23151). The cells were collected 3 h later, stained with DAPI and an anti-BrdU antibody, and the percentage of BrdU^+^ cells determined.

### Fluorescence and light microscopy

Eyes were enucleated and fixed for 20 min in cold 4% paraformaldehyde in phosphate-buffered saline (PBS) [65]. Retinas were dissected in PBS and incubated overnight at 4 °C in TNBT (10 mM Tris HCl, pH 7.4, 150 mM NaCl, 3% blocking reagent (ThermoFisher, 37580), 0.5% Triton X-100). Retinas were incubated overnight at 4 °C with IsoB4 and the indicated primary antibodies diluted in blocking buffer. The next day, retinas were washed three times in PBS and incubated with the corresponding secondary antibody for 2 h at room temperature (RT). Retinas were further washed and flat-mounted on glass slides using the FluorSave medium (Millipore, 345789).

HUVECs were seeded on 14 mm-diameter fibronectin-coated cover slides, starved for 3 h in endothelial cell basal medium (EBM-2) supplemented with 0.5% fetal bovine serum (FBS), and further treated with 10 nM chemerin and/or 100 µM CoCl_2_ in the same medium for 24 h. The cells were fixed with cold 4% paraformaldehyde for 15 min, permeabilized by 0.1% Triton X-100 for 10 min, and blocked in 3 % bovine serum albumin (BSA, AmericanBIO, AB01088-00100). They were further incubated overnight at 4 °C with the indicated primary antibodies followed by 1 h incubation with appropriate secondary antibodies at RT.

Frozen sections were fixed in acetone for 10 min, washed in PBS, and blocked for 30 min with 5% BSA in PBS. The sections were incubated overnight with the indicated primary antibodies at 4 °C, washed and incubated with secondary antibodies for 2 h at RT.

Eyes were enucleated and fixed overnight in Davidson’s fixative. After paraffin embedding, the eyes were cut into 5 μm sections and stained with hematoxylin and eosin. The thickness of the outer nuclear layer (ONL) was quantified with the Fiji software.

### Western blotting

Total lysates from HUVECs were obtained by lysing the cells in RIPA buffer containing protease (Roche, 11836145001) and phosphatase (Sigma, 4906845001) inhibitors. The protein concentration was measured by the bicinchoninic acid (BCA) assay (Bio-Rad). Equal amounts of proteins were separated on NuPAGE 4–12% gradient Bis-Tris gels (ThermoFisher, NW04125BOX) and transferred onto nitrocellulose membranes. After blocking with nonfat dry milk, the membranes were incubated overnight at 4 °C with the indicated primary antibodies. An anti-a1 actin antibody was used as loading control. Following a 2-h incubation at room temperature with a horseradish peroxidase-conjugated antibody, bands were visualized by chemiluminescence using the Plus-ECL substrate (Perkin Elmer) and a Fusion Solo S imaging system (Vilber). The intensity of the bands was quantified by the Fiji software.

### Tube formation assay

Tube formation assays were performed in µ-Slide Angiogenesis wells (Ibidi) using 10 µl of growth factor-reduced Matrigel (Corning, 356231) per well. Briefly, HUVECs were starved overnight in EBM-2 supplemented with 0.5% FBS, resuspended in the same medium containing 30 ng/ml VEGF (Peprotech, 450−32), seeded onto the polymerized Matrigel, and incubated for 5 h in a humidified chamber at 37 °C, 5% CO_2_. New medium containing 30 ng/ml VEGF and/or 10 nM chemerin was then added, and images of one field per well were acquired 3 and 9 h later. All conditions were run in duplicates and the number of tubes was quantified manually in six independent experiments.

### Wound-healing assay

HUVECs were seeded in a Culture-Insert 2 Well (Ibidi) at a density of 21 × 10^3^ cells per insert. When confluents, the cells were starved for 6 h in EBM-2 supplemented with 0.5% FBS. The insert was then removed and fresh medium containing 30 ng/ml VEGF and/or 10 nM chemerin was added. After 24 h, cells were fixed with cold 4% PFA, stained with DAPI, and images of one field per well were acquired. All conditions were run in duplicates and the number of cells that migrated into the gap were counted manually in three independent experiments.

### Aortic ring assay

Thoracic aortas were dissected from 3 mice per group and sectioned into segments (~ 500 μm long, > 6 segments per aorta), taking care not to damage the endothelium. The rings were embedded in Matrigel as previously described [33] and incubated in the presence of 30 ng/ml VEGF and various concentrations of recombinant mouse chemerin (0, 10, 20, and 50 nM). Each ring was photographed 5 days later. The area of endothelial sprouts surrounding the rings was analyzed with the Fiji software.

### Pharmacological treatments

For inhibiting the Notch pathway, the γ-secretase inhibitor DAPT (Sigma, D5942) was solubilized at 1 µg/µl in 10 mM phosphate-buffered saline (PBS) containing 5% dimethyl sulfoxide (DMSO) and injected intraperitoneally (0.1 mg kg^− 1^) at post-natal days 4 and 5. The FoxO1 inhibitor AS1842856 (2.5 mg kg^− 1^, Calbiochem, 344355) solubilized in saline containing 5% DMSO or the PTEN inhibitor bpV(HOpic) (2 mg kg^− 1^, Millipore, 203701) solubilized in saline were injected subcutaneously in the vicinity of the eye at post-natal days 3 and 5. The vehicles were used as controls. Mice were sacrificed at day 6 and the retinas collected.

### In vivo EdU labeling and detection

5-Ethynyl-2′-deoxyuridine (EdU, 100 µg/g body weight) was injected i.p. into P6 pups 2.5 h before sacrifice. EdU^+^ cells in the retina were detected with the Click-iT EdU Alexa Fluor 488 Imaging Kit (ThermoFisher, 10337) according to the manufacturer’s instructions. ECs were counterstained with IsoB4 and their nuclei by an anti-ERG antibody.

### Oxygen-induced retinopathy (OIR) model

As previously described [34], pups were placed from P7 to P12 in 75% oxygen (hyperoxia) and returned at P12 to normal oxygen conditions (normoxia) until P17. Retinas were collected at P12 and P17.

### Hind-limb ischemia model

Mice underwent unilateral femoral artery ligation as described [66]. Briefly, following anesthesia, the left femoral artery was exposed under a dissecting microscope. The proximal femoral artery and the distal portion of the saphenous artery were ligated, and an arteriectomy was performed. Blood flow was measured at day − 1, 0, 4, 7, 14, and 21 using a laser Doppler imaging system (Moor Instruments). On day 21, the mice were sacrificed, and the left and right gastrocnemius muscles were harvested, embedded in Tissue-TEK OCT compound, and frozen for cryosectioning.

### Tumor models and imaging

Control and K5-Chem mice (6 to 8 weeks old) were injected subcutaneously with 10^6^ Lewis lung carcinoma (LLC) or B16-F0 melanoma cells (American Type Culture Collection). The size of the tumors was monitored with a caliper, and LLC and B16 tumors were harvested and weighed after, respectively, 13 and 9 days. Tumors were either embedded in Tissue-Tek OCT compound or processed through the iDISCO protocol [67] for tissue clearing. Briefly, tumors were fixed for 24 h in cold 4% paraformaldehyde, dehydrated in an ascending methanol/water series (20 to 100%, 1 h each), transferred overnight in dichloromethane/methanol (2:1 v/v), and bleached overnight at 4 °C in methanol containing 5% H_2_O_2_ (Sigma, D216763). After rehydration through a methanol/water series (100–20%, 1 h each) and transfer to PBS containing 0.2% Triton X-100, the samples were incubated overnight at 37 °C in PBS containing 0.2% Triton X-100, 20% DMSO, and 0.3 M glycine (Sigma, G8898), then overnight at 37 °C in a blocking solution (PBS containing 0.2% Triton X-100, 10% DMSO, and 6% donkey serum), and washed overnight in PBS containing 0.2% Tween-20 (Sigma, P9416) and 10 mg/ml heparin (PTwH). The tumors were then incubated with primary antibodies diluted in PTwH containing 5% DMSO and 3% goat serum, for 5 to 6 days at 37 °C with gentle shaking on an oscillator, refreshing the primary antibodies once after 3 to 4 days. Tumors were washed four times for 1 h in PTwH, incubated for 3 to 4 days at 37 °C with gentle shaking with secondary antibodies diluted in PTwH containing 3% goat serum, and washed five times for 1 h in PTwH. Following dehydration through an ascending methanol/water series and incubation for 3 h in dichloromethane/methanol (2:1 v/v), the samples were incubated twice for 15 min in dichloromethane and finally in dibenzyl ether (Sigma, 108014). Imaging of the entire tumor was performed on a selective plane illumination microscope (Ultramicroscope II, LaVision Biotec) at 2.5× magnification, with a 25 μm step between tiles.

### Morphometric analyses

Confocal images were acquired on a Zeiss LSM 780 NLO system fitted on an Axio Observer Z1 inverted microscope (Zeiss) equipped with a Chameleon Vision II 690–1064 nm multiphoton laser (Coherent Europe). Tiles were imaged with an Axio Imager Z1 fluorescence microscope (Zeiss).

For the quantification of vessels density, total vessels length, and branching points, images of the IsoB4-labeled capillary plexus were obtained with a 20× objective for each leaflet, in the remodeling zone and the proliferative zones of the network. The images were analyzed with the Angiotool software [68]. For each parameter, the mean of all values obtained for a retina was recorded as a data point.

Radial expansion was the distance between the optic disc and the network front, divided by the distance between the optic disc and the retina edge, determined on a reconstruction of the whole IsoB4-stained retina from tiles obtained with a 20x objective. The mean of values obtained for all leaflets of a retina was recorded as a data point.

The number of ESM1^+^ ERG^+^ cells was counted in Fiji on the leading edge of the network, on reconstructed images (20x) of the whole retina stained with IsoB4 and for ESM1 and ERG, and divided by the length of the angiogenic front. The mean per retina was recorded as a data point. EC proliferation was estimated by counting the proportion of EdU^+^ nuclei among ERG^+^ nuclei on images (20×) taken in the proliferative zone of retinas stained with IsoB4 and for ERG and EdU. The mean per retina was recorded as a data point.

Pericyte coverage was determined in Fiji from images (20×) taken in the remodeling plexus, by dividing the area stained for α-SMA, desmin or PDGFRβ by the surface stained by IsoB4. The mean from the four retina leaflets was recorded as a data point.

For the quantification of basal membrane empty sleeves and apoptototic cells, reconstructed images (20×) of whole retinas stained with IsoB4, and for ColIV and cCasp3, were analyzed in Fiji. The number of empty sleeves (ColIV^+^ and IsoB4^−^) and cCasp3^+^ cells were counted in the areas surrounding main arteries and first order arterial branches, or main veins and first order vein branches, in the remodeling and proliferative zones, and divided by the respective surface. Arteries and veins in damaged areas were excluded. For each parameter, the mean of all values obtained for a retina was recorded as a data point.

For the OIR model, IsoB4-stained retinas were imaged (20×) and reconstructed. The avascular area and surface of neovascular tufts were quantified in Fiji and divided by the total retinal area.

Frozen sections from tumors or gastrocnemius muscle, stained by DAPI and for CD31 and/or ColIV, were imaged (20×) as tiles and reconstructed. The CD31^+^ area, normalized to the total surface of the tissue, was measured in Fiji. For tumors, the ColIV^+^ area was measured and normalized to the CD31^+^ area. Two to three sections per sample were analyzed and the mean per mouse was recorded as a data point.

### Statistics and artwork

All data are presented as mean ± SEM. Statistical analysis was performed using the Instat or GraphPad Prism 7 softwares. The unpaired two-tailed Student’s *t*-test was used to determine statistical significance between two groups. For comparison of more than two groups, we performed one-way or two-way ANOVA followed by Tukey’s post hoc test. Reproducibility was ensured by performing several independent experiments. Analyses were performed blinded, without knowing the genotype of animals, the treatment received, or experimental conditions. All experimental data collected from transgenic or knockout animals were compared with those obtained from littermate controls. For the retinal parameters and OIR experiments, the body weight of mice was measured, and outliers that were over two standard deviations away from the mean of their littermates were excluded. In all experiments, animals were age-matched and distributed randomly into groups. Graphs were generated in GraphPad Prism 7 and figures assembled in Adobe Illustrator.

## Electronic Supplementary Material

Below is the link to the electronic supplementary material.

Supplementary file1 (AVI 29769 kb) Z‐stack projection of the retina of an adult control mouse stained with the vascular marker Isolectin B4 (Red) and for the smooth cell marker αSMA (Green).

Supplementary file1 (AVI 29646 kb) Z‐stack projection of the retina of an adult K5-Chem mouse stained with the vascular marker Isolectin B4 (Red) and for the smooth cell marker αSMA (Green).

Supplementary file1 (AVI 29152 kb) 3D reconstruction of a cleared LLC tumor collected at day 13 post-graft from a control mouse, stained with the vascular marker Isolectin B4 (Red) and for the basal lamina marker collagen IV (Green).

Supplementary file1 (AVI 29028 kb) 3D reconstruction of a cleared LLC tumor collected at day 13 post-graft from a K5-Chem mouse, stained with the vascular marker Isolectin B4 (Red) and for the basal lamina marker collagen IV (Green).

## Data Availability

The datasets generated during and/or analyzed during the current study are available from the corresponding author on reasonable request.
